# Extrapulmonary Coinfection Caused by Pneumocystis jirovecii and Histoplasma capsulatum in an Adult With Human Immunodeficiency Virus Infection: A Case Report

**DOI:** 10.7759/cureus.76808

**Published:** 2025-01-02

**Authors:** Julián Rondón-Carvajal, Manuela Gil-González, Sebastián Ruiz-Giraldo, Miguel Pinzón, Juan Carlos Gómez-Velásquez

**Affiliations:** 1 Internal Medicine - Pulmonology, Corporación en Estudios de la Salud (CES) University, Medellín, COL; 2 General Medicine, Faculty of Medicine, University of Antioquia, Medellín, COL; 3 Infectious Diseases, Hospital San Vicente Fundación, Rionegro, COL; 4 Infectious Disease, Clinica Medellin, Medellín, COL; 5 Medical Microbiology, SYNLAB Colombia, Medellín, COL

**Keywords:** acquired immune deficiency syndrome (aids), acute miliary histoplasmosis, extrapulmonary pneumocystosis, human immunodeficiency virus (hiv) infection, molecular diagnosis of infectious diseases

## Abstract

Pulmonary coinfection by *Pneumocystis jirovecii* and *Histoplasma capsulatum* in patients with human immunodeficiency virus infection and acquired immune deficiency syndrome (HIV/AIDS) is common. However, coinfection by extrapulmonary pneumocystis and disseminated histoplasmosis is not. We report a 33-year-old Colombian male patient with a recent diagnosis of HIV/AIDS infection presented with mild flu-like symptoms, chronic diarrhea, cachexia, pale conjunctiva, oral ulcers, and painful hepatomegaly for about 15 days. Pancytopenia without jaundice was documented. Computed tomography showed ground-glass and micronodular miliary patterns suggestive of *P. jirovecii* pneumonia, pulmonary involvement due to miliary tuberculosis, or histoplasmosis. Histological samples of bronchoalveolar lavage and laparoscopic liver biopsy revealed structures of *P. jirovecii*, which are verified by polymerase chain reaction. Histoplasma urine antigen was positive. *H. capsulatum* infection was confirmed by fungal isolation from blood culture and matrix-assisted laser desorption ionization time of flight mass spectrometry. The patient was treated with clindamycin, oral primaquine, and intravenous amphotericin B plus maintenance therapy with itraconazole, and the clinical response was excellent. This case report highlights that despite effective, highly active antiretroviral therapy (ART), rare instances of extrapulmonary coinfection by *P. jirovecii* and *H. capsulatum* can still occur. It is therefore important to have a high suspicion index of extrapulmonary pneumocystosis and initiating treatment to prevent mortality. Factors such as severe immunosuppression (CD4+ T-lymphocyte counts <40/mm³) in patients with undiagnosed HIV, treatment-naïve individuals, those who discontinue ART and the absence of *P. jirovecii* prophylaxis may increase clinicians' suspicion of extrapulmonary manifestations in HIV-infected patients.

## Introduction

Pneumocystosis and Histoplasmosis are infections caused by the opportunistic fungi *Pneumocystis jirovecii* and *Histoplasma capsulatum*, respectively. *P. jirovecii* is an extracellular microorganism that causes life-threatening pneumonia mainly in people living with human immunodeficiency virus infection and acquired immune deficiency syndrome (HIV/AIDS), especially with a CD4+ T-lymphocyte count (CD4) below 200/uL [1]. Annually, the incidence of *P. jirovecii* pneumonia (PJP) in these people exceeds 400,000 cases worldwide, and the fatality rate is approximately 10%-30% [2]. Extrapulmonary and disseminated infection by this fungus is remarkably uncommon, and it is associated with a high mortality rate occurring mostly in patients with highly advanced HIV infection (CDC stage 3 or acquired immunodeficiency syndrome) [3]. The diagnosis of *P. jirovecii* infection is based on visualization of the ascii and/or trophic forms in clinical samples using special stains or by the amplification of specific genes using polymerase chain reactions (PCR). Also, the 1-3-β-d-glucan assay is used to support the *P. jirovecii* infection diagnosis, although it is not very specific for this agent, being positive for other fungal infections [4]. Trimethoprim-sulfamethoxazole (TMP-SMX) is the first-line regimen for the treatment of pneumocystosis [5].

On the other hand, *H. capsulatum* is a dimorphic fungus that causes endemic mycoses known as histoplasmosis, mainly in tropical regions of Latin American countries [6]. The disease begins when conidia or small hyphal elements are inhaled and converted into blastoconidia forms in the lungs or when organisms in previous quiescent foci of infection are reactivated during immunosuppression [7]. Immunocompromised patients such as those with HIV/AIDS frequently have disseminated infection involving the liver, skin, spleen, gastrointestinal tract, and bone marrow, with an incidence of 1.5 per 100 person-years [8]. The approach to diagnosis histoplasmosis includes visualization of the blastoconidia cells by cytology or histopathology using specific stains, antigen detection in urine and/or serum, or PCR-based methods for the detection of the fungus; definitive diagnosis relies upon the isolation and identification of *H. capsulatum* [9,10]. Liposomal amphotericin B is the preferred treatment for severe cases of disseminated histoplasmosis treatment [9]. *P. jirovecii* and *H. capsulatum* pulmonary coinfection has been described. However, to our knowledge, extrapulmonary coinfection has not been reported. Herein, we present the case of an HIV/AIDS Colombian patient who developed both hepatic pneumocystis infection and disseminated histoplasmosis.

## Case presentation

A 33-year-old Colombian male patient with HIV/AIDS diagnosis, characterized by a CD4 cell count of 28 cells/mm³ and a plasma HIV RNA level of 566.357 copies/mL, started antiretroviral therapy (ART) with zidovudine combined with lamivudine and efavirenz. Thirty days after the HIV/AIDS diagnosis and the start of the therapy (day 0), the patient complained of asthenia, fatigue, odynophagia, headache, dizziness, nausea, inappetence, diarrhea, and cough with expectoration of white sputum. These symptoms had persisted for the previous 15 days. No alteration of vital signs was registered. Physical examination revealed cachexia, pale conjunctiva, ulcerative lesions of the oral mucosa, and painful hepatomegaly without jaundice. No other physical abnormalities were identified. Laboratory tests were requested, and the results thus obtained are shown in Table 1.

**Table 1:** Admission laboratory tests results ALT: alanine aminotransferase; AST: aspartate aminotransferase; ALP: alkaline phosphatase

Parameter	Value	Reference value	Parameter	Value	Reference value
Hemoglobin	8.0 g/dL	11.4 g/dL	ALT	153 U/L	4-41 U/L
White blood cells	3,570 cells/mm^3^	4,500-11,000 cells/mm^3^	AST	108 U/L	0-39 U/L
Neutrophil	81.3%	40.3%-74.8%	ALP	125 U/L	Until 125 U/L
Platelet count	140 × 10^3^/µL	150-400 × 10^3^/µL	Total bilirubin	0.92 mg/dL	0.3-1 mg/dL
Creatinine	0.97 mg/dL	0.6-1.2 mg/dL	Conjugated bilirubin	0.42 mg/dL	0.1-0.3 mg/dL

Blood cultures for fungi, mycobacteria, and anaerobic and aerobic bacteria were drawn. *H. capsulatum* urine antigen, serum Aspergillus galactomannan, and Cryptococcus spp. antigen were requested. A stool wet mount microscopic examination, modified Ziehl-Neelsen (ZN) stain for fecal smears, and stool culture were carried out. Parasites and Cryptosporidium spp. were not observed, nor were enteropathogenic bacteria isolated. The patient had oral ulcers clinically compatible with herpetic stomatitis. He was not receiving TMP-SMX for PJP prophylaxis. Therefore, treatment with intravenous (IV) acyclovir 500 mg every eight hours was initiated for seven days. Additionally, PJP prophylaxis was started with IV TMP-SMX 160/800 mg daily.

**Figure 1:**
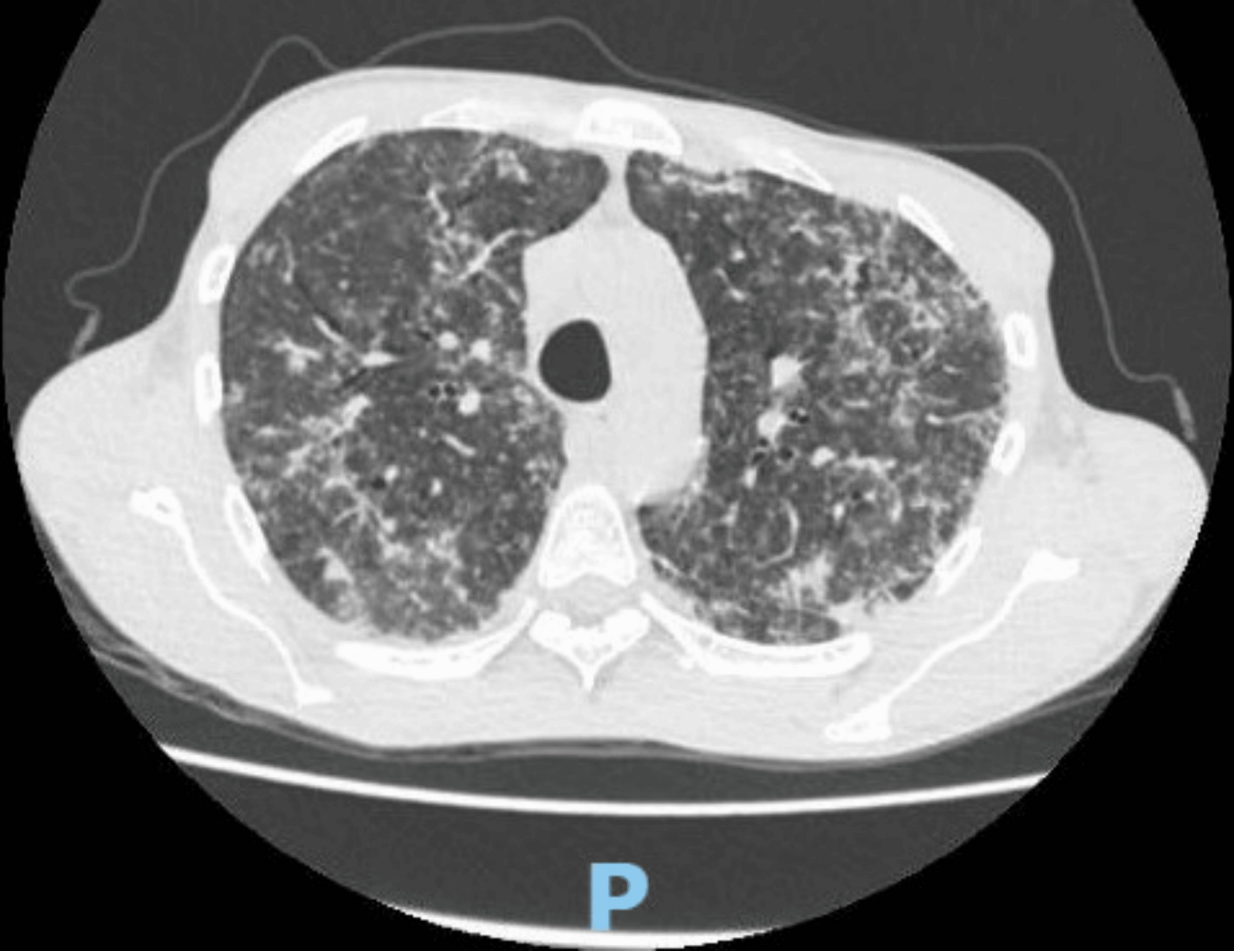
Axial section of chest contrast-enhanced CT scan Innumerable, small 1-4 mm pulmonary nodules scattered throughout the lungs. Additionally, some miliary opacities are very dense, narrowing the differential diagnosis CT: computerized tomography

On day 1, a local allergic reaction appeared, prompting the replacement of PJP TMP-SMX prophylaxis with oral dapsone 50 mg/dose and pyrimethamine 25 mg/dose weekly. Additionally, calcium folinate therapy was added. At this time, the patient underwent a colonoscopy and a contrast-enhanced computed tomography (CT) of the head, which revealed no abnormalities. However, chest contrast-enhanced CT showed a ground-glass and micronodular miliary pattern (Figure 1), suggesting both PJP and either histoplasmosis or milliary tuberculosis. Therefore, a ZN stain was requested on a clinical sample obtained via bronchoalveolar lavage (BAL). On day 2, PJP prophylaxis was discontinued due to chest contrast-enhanced CT findings. Treatment with IV clindamycin 600 mg every eight hours and oral primaquine 30 mg/day therapy was then started. Additionally, because of the high suspicion of histoplasmosis, IV amphotericin deoxycholate B 50 mg/day was administered. Due to low hemoglobin levels (8.0 g/dL), zidovudine/lamivudine was withdrawn, and emtricitabine/tenofovir 200 mg/300mg plus efavirenz 600 mg/day was started. Blood chemistry revealed a high level of liver enzymes ×3 times the upper limit of normal (alanine aminotransferase 153 U/L, aspartate aminotransferase 108 U/L) and alkaline phosphatase ×5 (ALP 688 U/L), raising suspicion of hepatic infiltration (Table 2). Consequently, a laparoscopic liver biopsy was performed, and the samples were sent for histological assessment, Gram staining, direct potassium hydroxide examination, ZN stain, and modified Kinyoun stain for Nocardia spp. Cultures for aerobic bacteria, fungi, and *Mycobacterium tuberculosis* were also performed. Additionally, the molecular genetic test GeneXpert MTB/RIF (Ultra) was used for *M. tuberculosis* DNA detection.

**Table 2:** Liver biochemistry profile at day 2 ALT: alanine aminotransferase; AST: aspartate aminotransferase; ALP: alkaline phosphatase

Parameter	Value	Reference value
ALT	153 U/L	4-41 U/L
AST	108 U/L	0-39 U/L
ALP	688 U/L	Until 125 U/L

On day 3, the HIV viral load was quantified, and clinical samples for hepatitis B virus, hepatitis C virus, and cryptococcal capsular antigen detection were obtained. On day 4, sputum ZN staining was performed, and no acid-fast bacilli were observed. With this result and considering clinical improvement, treatment for tuberculosis was not recommended, and suspicion of histoplasmosis increased. On day 5, the results of the aerobic bacteria culture on the liver tissue were negative. On day 6, the Platelia™ Aspergillus assay (Bio-Rad, Marnes-la-Coquette, France) was performed for Aspergillus galactomannan serum detection, according to the manufacturer’s instructions. The result of the test was 3.27 (cutoff index >0.5 is reported as positive).

On day 8, the HIV viral load was measured and showed a significant decrease to 74 copies/mL, compared to the initial value of 566,357 copies/mL. Hepatitis B surface antigen was measured at 0.51 cutoff index (COI; reference range: 0-0.89 COI), and the immunoglobulin M antibody to hepatitis B core was at 0.08 COI (reference range: 0-0.99 COI). The hepatitis B DNA PCR test result was less than 117 international units (IU)/mL (reference range: 0-117 IU/mL). The antibody test for hepatitis C showed 0.05 COI (reference range: 0-0.89 COI). Based on these results, hepatitis B and C infections were ruled out. Additionally, the cryptococcal antigen test yielded negative results. The level of creatinine increased to 1.34 mg/dL, which was assumed to be due to amphotericin B nephrotoxicity. On day 11, the patient presented acute kidney injury (creatinine: 1.6 mg/dL). Consequently, amphotericin B was infused over 24 hours, and renal function improved by day 14. On day 15, BAL was obtained via fiberoptic bronchoscopy to detect M. tuberculosis, fungi, or bacteria.

**Figure 2:**
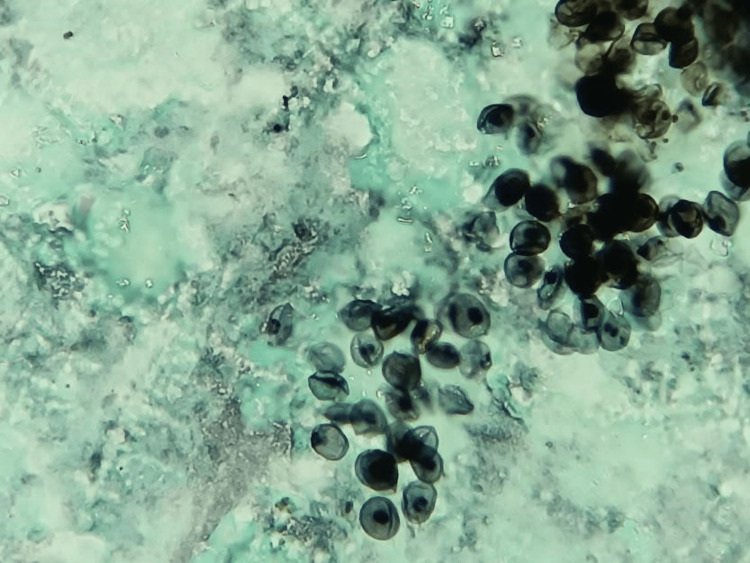
BAL sample Cyst-like structures (ascii) of *P. jirovecii* in BAL sample staining with GMS (400×) BAL: bronchoalveolar lavage; GMS: Gomori methenamine silver

**Figure 3:**
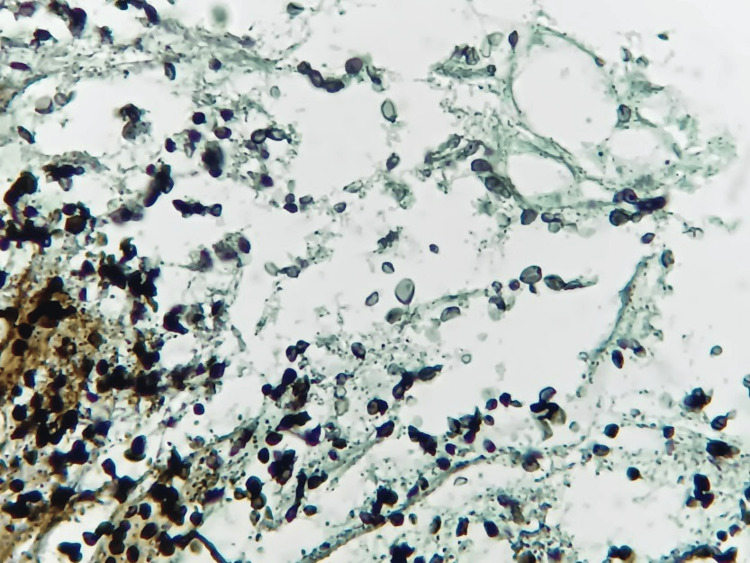
Sample taken through liver biopsy Cyst-like structures (ascii) compatible with *P. jirovecii* in liver tissue (400×)

On day 16, microbiological results of staining for bacteria and molecular tests for *M. tuberculosis* in BAL and liver tissue were negative, but Gomori methenamine silver (GMS) staining revealed cyst-like (ascii) structures of *P. jirovecii* in the BAL sample (Figure 2). This finding was confirmed by PCR. Furthermore, cystic-like structures (ascii) of *P. jirovecii *were observed in liver tissue stained with GMS (Figure 3). Additionally, Histoplasma urine antigen (IMMY, Norman, OK, USA) was detected.

On day 17, the patient showed significant improvement in clinical and laboratory outcomes, leading to his discharge home. For outpatient treatment, amphotericin B therapy was replaced by oral itraconazole 200 mg every 12 hours until the CD4 cell count exceeds 200/mL. Additionally, clindamycin 600 mg orally every eight hours and primaquine 30 mg/day orally were prescribed for another five days. Furthermore, primary prophylaxis against PJP was prescribed with TMP-SMX 160/800 mg orally three times a week, along with loratadine 10 mg orally daily to mitigate mild allergies.

**Figure 4:**
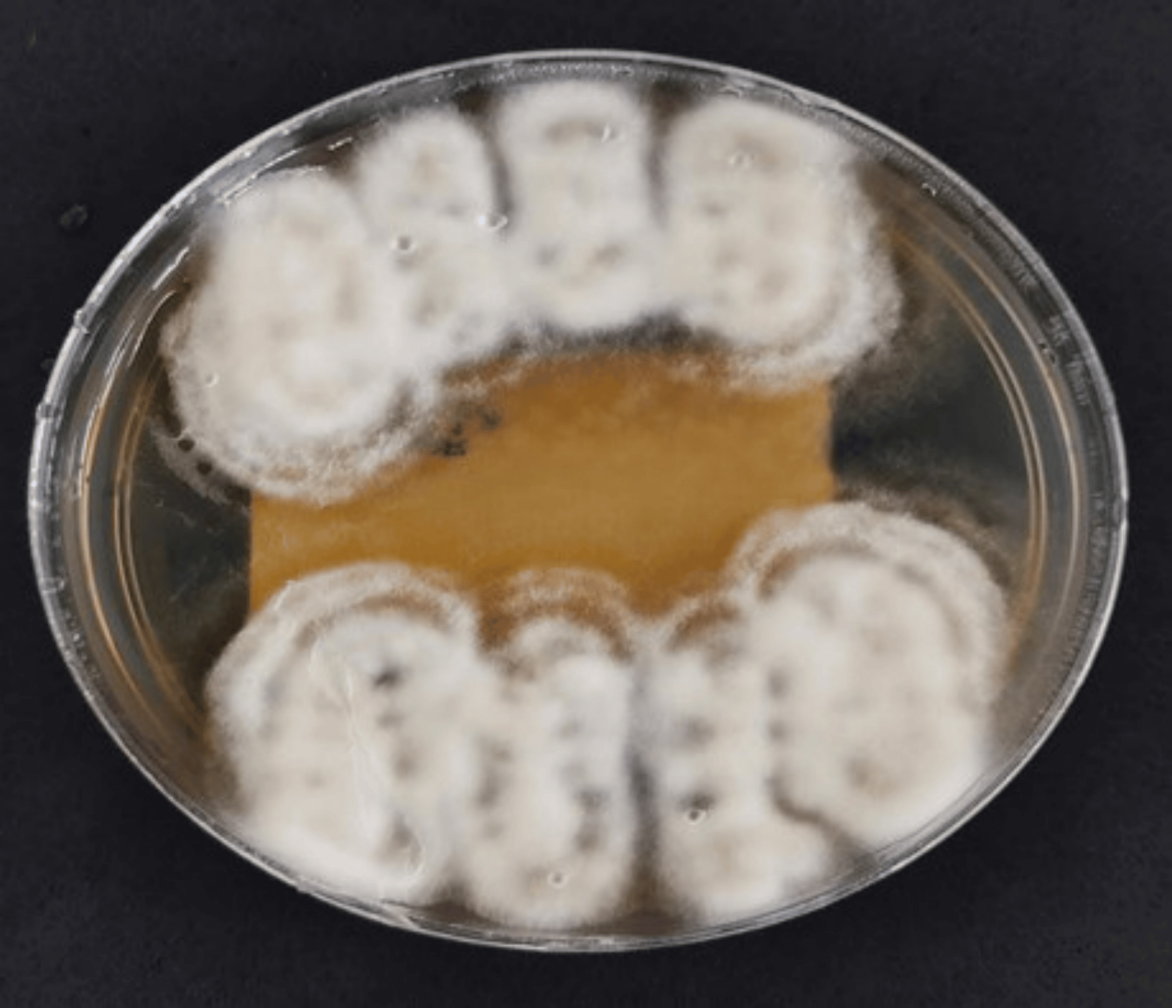
BHI-cysteine-blood agar at 37°C Colonies of *H. capsulatum* isolated from blood culture BHI: brain heart infusion

**Figure 5:**
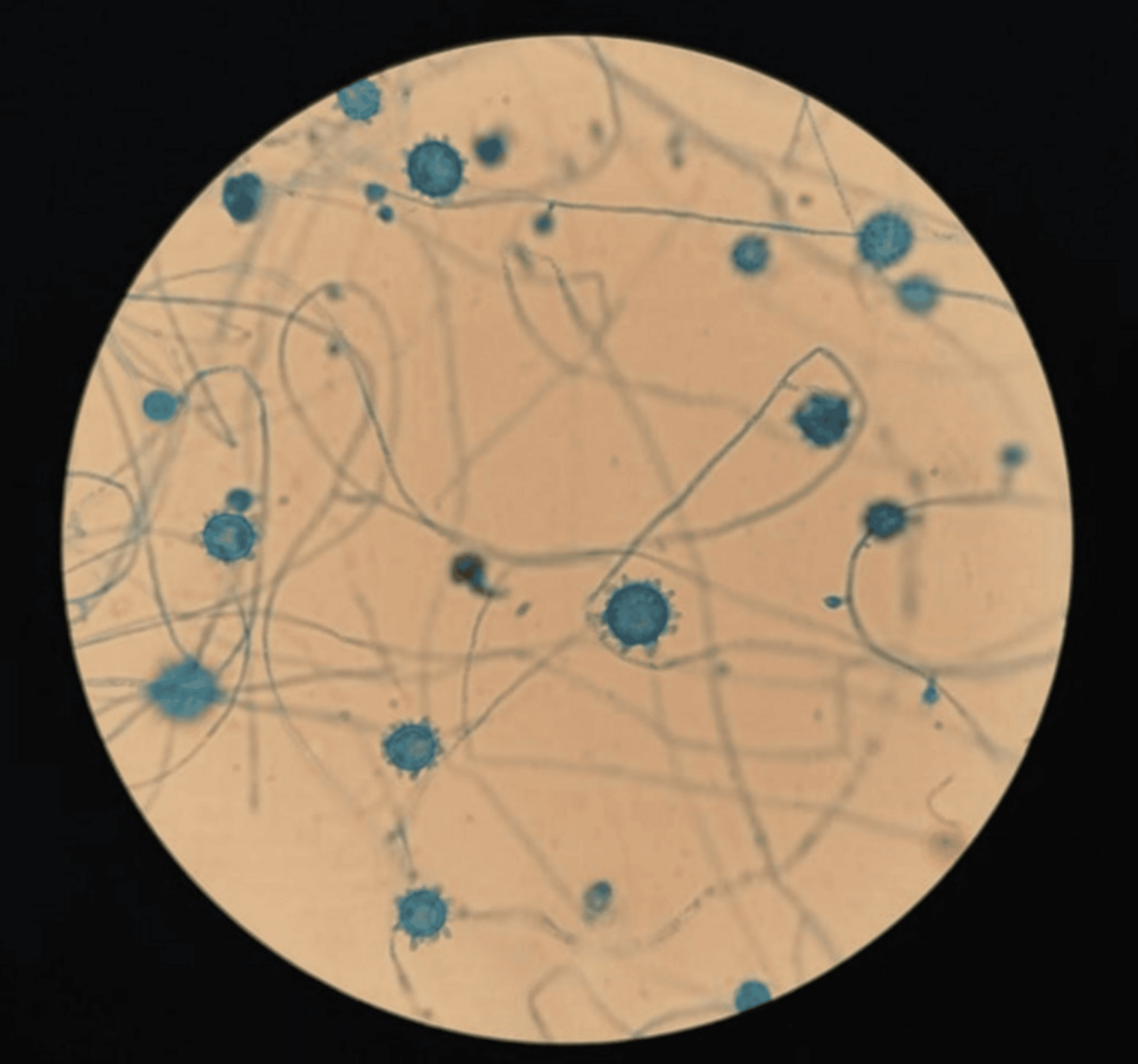
H. capsulatum, microscopic appearance with LPCB test (magnification 400×) Tuberculate macroconidia and microconidia of *H. capsulatum*. Characteristic macroconidia are large, thick-walled, round, typically tuberculate, or knobby (7-15 μm in diameter). Microconidia are smooth-walled spherical, pyriform (2-5 μm in diameter) on short branches or directly on the sides of the hyphae LPCB: lactophenol cotton blue

On day 42, the *H. capsulatum* infection was confirmed through fungal isolation from blood cultures (Figures 4, 5). The identification of *H. capsulatum* was further confirmed by matrix-assisted laser desorption ionization time of flight mass spectrometry. The patient recovered from his infection; however, follow-up was discontinued after he moved to a different medical center for social security reasons.

## Discussion

Various cases of pulmonary coinfection by *P. jirovecii* and *H. capsulatum* in patients with HIV/AIDS have been published [10,11]. However, to date, no cases of extrapulmonary coinfection have been reported by these two pathogens. In this report, we describe the case of a patient living with HIV/AIDS presenting with an extrapulmonary coinfection by *P. jirovecii* and *H. capsulatum* affecting the liver and bloodstream, respectively. *P. jirovecii* was detected in BAL samples and liver tissue, while *H. capsulatum* was isolated from a blood culture. This is the first reported case of extrapulmonary pneumocystosis in Colombia.

Extrapulmonary infection, dissemination, and coinfection represent uncommon and distinct clinical manifestations of *P. jirovecii* infections, delineating the scope of the infection, particularly in immunocompromised patients. Due to their rarity, there is no consensus on these clinical concepts. However, current evidence suggests that extrapulmonary infection indicates the presence of *P. jirovecii* outside the lungs, regardless of any pulmonary involvement. Disseminated infection, on the other hand, describes the spread of *P. jirovecii* to various organs or body areas, indicating a widespread infection that affects multiple sites, even if not confirmed by a blood smear. Coinfection refers to the simultaneous presence of *P. jirovecii* and another pathogen. Sometimes, the terms extrapulmonary and disseminated infections are used interchangeably. Table 3 presents the cases of extrapulmonary, disseminated infections, and extrapulmonary coinfections by *P. jirovecii* in both HIV-infected patients and those with non-HIV-related immunodeficiency reported in the literature from 2014 to 2024.

**Table 3:** Case reports of extrapulmonary/disseminated infections and coinfections caused by P. jirovecii in both HIV-infected patients and those with non-HIV-related immunodeficiency reported in the literature from 2014 to 2024 HIV: human immunodeficiency virus; CD4: CD4+ T-lymphocyte count; CTLA-4: cytotoxic T-lymphocyte antigen 4

Year	Study	Age	Immunological condition	Extrapulmonary infection	Extrapulmonary coinfection
2014	Karam and Mosadegh [10]	46	HIV (CD4 1.8/mm³)	Spleen, vertebral lesion	No
2015	Valdebenito et al. [11]	41	HIV (CD4 35/mm³)	Colon	No
2016	Boontanondha and Kiertiburanakul [12]	22	Adult-onset immunodeficiency positive for interferon-gamma autoantibody	Cervical lymph node	Disseminated *Mycobacterium abscessus*
2018	Radisic et al. [13]	30	HIV (CD4 35/mm³)	Retroperitoneal lymph nodes	No
2019	Righi et al. [14]	58	Corticosteroids, cyclophosphamide, and rituximab	Blood	No
2020	Siddiqi et al. [15]	24	CTLA-4 haploinsufficiency	Bone	No
2020	Abbas et al. [16]	31	HIV (CD4 <20/mm³)	Spleen	No
2021	Filippidis et al. [17]	31	HIV (CD4 3/mm³)	Thyroid gland, lymph nodes	No
2022	Hasegawa et al. [18]	39	HIV (CD4 36/mm³)	Spleen	No
2023	Kojima et al. [19]	45	HIV (CD4 14/mm³)	Spleen	No
2023	Tancharoen et al. [20]	45	HIV (CD4 16/mm³)	Paravertebral mass	No

Extrapulmonary pneumocystosis predominantly affects HIV-infected individuals, accounting for the majority of cases (91.6%), as reported by Valdebenito et al. [11]. This type of infection is usually a manifestation of HIV disease progression in patients who have not yet been diagnosed with HIV or who have poor adherence to ART. While immunodeficiency due to HIV infection is the most frequent, extrapulmonary or disseminated infection by *P. jirovecii* can also occur in individuals with adult-onset immunodeficiency, cytotoxic T-lymphocyte antigen 4 haploinsufficiency disorders, secondary immunodeficiency due to immunosuppressive therapy, or even immunocompetent adults [12,13].

Previous case reports indicate that extrapulmonary Pneumocystis infection can involve multiple sites. In order of frequency, these sites include the spleen, lymph nodes, vertebral tissue, bowel, adrenal glands, bone, bloodstream, and thyroid gland (Table 3). Additionally, cases have been documented in the skin, brain, ear, skeletal tissue, and intra-abdominal mass walls [14]. Typically, infection with *P. jirovecii* affects the lungs. However, there are atypical cases of extrapulmonary infection without evidence of lung compromise or where it begins in the lungs and subsequently spreads to other sites, as observed in our case [13-15]. Autopsy findings have revealed that the infection spreads through hematogenous routes, lymphatic pathways, or direct extension from a nearby organ, as seen in cases of subpleural infection [15].

We present a case of a patient living with HIV/AIDS whose initial RNA HIV viral load was 566,357 copies/mL at diagnosis. Following the initiation of ART, this decreased to 74 copies/mL. Although such a reduction in viral load might suggest immune reconstitution inflammatory syndrome (IRIS), no systemic inflammatory response was observed. Our case contrasts with the IRIS linked to splenic *P. jirovecii* infection reported by Kojima et al. [19].

In this report, the patient presented with respiratory symptoms and a ground-glass pattern highly suggestive of PJP. However, due to the rarity of *P. jirovecii* spreading beyond the lungs to affect the liver, such involvement was not initially considered. Liver injury characterized as mixed by calculating the R factor (3.7) and painful hepatomegaly, as observed in the case described here, are clinical findings that can manifest in various infectious (bacterial, parasitic, and viral) or malignant diseases [16]. Consequently, suspecting an extrapulmonary *P. jirovecii* infection involving the liver presents a significant challenge for clinicians. This is particularly evident due to the rarity of liver infection by this fungus and the limited documentation available, with only one microbiologically confirmed case reported in the past 24 years [17]. Hepatic involvement in disseminated Histoplasmosis is common and would be the main clinical possibility in this case; however, no blastoconidia forms compatible with *H. capsulatum* were observed, and tissue culture was negative for this fungus. Instead, tissue stains demonstrated *P. jirovecii* structures, confirming extrapulmonary infection.

Extrapulmonary pneumocystosis is diagnosed by identifying *P. jirovecii* cysts in affected tissues, often incidentally discovered by clinical microbiologists or pathologists, as in our case [17,18]. These professionals play a crucial role in establishing the diagnosis, recognizing the characteristic foamy eosinophilic material containing *P. jirovecii* cysts and trophozoites. Confirmation of diagnosis typically involves performing additional stains, if necessary; in our case, we utilized Gomori-methenamine-silver (GMS), consistent with prior documented approaches [18,19]. Giemsa staining and immunofluorescent techniques using monoclonal antipneumocystis antibodies have also been employed [20].

Given the diverse clinical manifestations of fungal infections in HIV/AIDS patients and the potential for infections by various fungi, particularly *H. capsulatum* and *P. jirovecii*, it is crucial to consider the possibility of mixed fungal infections, especially in regions endemic to *H. capsulatum*. According to Carreto-Binaghi et al. [9], coinfection with *H. capsulatum* and *P. jirovecii* is more prevalent than commonly assumed, even beyond immunocompromised populations. In our case, the utility of urinary antigen testing for the prompt diagnosis of histoplasmosis in immunocompromised patients is underscored, particularly given the lengthy culture time required for fungal growth. Other extrapulmonary coinfections identified in HIV-infected patients include *Mycobacterium abscessus*, Cryptococcus, and cytomegalovirus [12-16].

Multiplex real-time PCR or quantitative polymerase chain reaction multiplex [18-20] assays offer viable alternatives for promptly diagnosing fungal coinfections. It is important to note that while the Aspergillus galactomannan test yielded a positive result (3.27), this represents cross-reactivity with the galactomannan of *H. capsulatum* [18,19]. The patient had no clinical or microbiological evidence of aspergillosis. Ultimately, while the patient exhibited clinical suspicion of multiple infections, microbiological confirmation was only achieved for histoplasmosis and pneumocystosis. Nonetheless, considering the patient's high risk of deterioration and mortality, treatment for histoplasmosis could not be postponed, prompting the initiation of therapy without prior confirmation of the Histoplasma urine antigen. Conversely, although infection by *M. tuberculosis* was also suspected, tuberculosis treatment was deferred as the patient was responding well to amphotericin B.

TMP-SMX is typically the first-line treatment for *P. jirovecii* infection, with most patients in Table 3 receiving a 21-day course [17-20]. However, in cases of extrapulmonary involvement, some authors have extended treatment for up to six weeks [20]. In our case, due to mild allergic reactions, clindamycin combined with primaquine was considered a successful alternative, resulting in an excellent treatment response, consistent with findings from other documented reports.

## Conclusions

This case report highlights that despite effective highly active ART, rare instances of extrapulmonary coinfection by *P. jirovecii* and *H. capsulatum* can still occur. Emphasis is placed on promptly considering extrapulmonary pneumocystosis and initiating treatment to prevent mortality. Factors such as severe immunosuppression (CD4 counts <40/mm³) in patients with undiagnosed HIV, treatment-naïve individuals, those who discontinue ART, and the absence of *P. jirovecii* prophylaxis may increase clinicians' suspicion of extrapulmonary manifestations in HIV-infected patients.
